# Progress in Fevers

**Published:** 1904-01-16

**Authors:** 


### Progress in Fevers.

(*Continued from page* 208.)

**Typhoid Fever.**—In a lecture[8] delivered at St. Thomas's Hospital, Hector Mackenzie deals at some length with the subject of perforation occurring during the course of typhoid fever. It is calculated that in this country in general practice perforation occurs once in about every 30 cases of typhoid fever and is the cause of the fatal termination in one out of every three or four deaths. Statistics dealing with 2,533 cases of typhoid treated in four large London hospitals give a mortality, from all causes, of 13·4 per cent., and a mortality from perforation of 4·5 per cent. A study of 5,878 cases admitted into the Metropolitan Asylums Board hospitals during 1897–1901 gives percentages of 15·6 and 3·2 per cent. respectively. It is suggested that the lower percentage observed in the latter may be due to the fact that post-mortem examinations are less constantly made in these hospitals than in general hospitals. Abroad the general typhoid mortality is much lower, less than half what it is in the London hospitals. Thus in 3,351 cases treated in the Johns Hopkins, the Montreal Royal Victoria, and the Brisbane hospitals, in all of which the cold bath treatment is employed, the general mortality works out at 7·1 per cent. and the perforation mortality at 2·7 per cent. It is to be observed that the proportion of perforation cases to fatal cases is about the same, or somewhat higher. In England 25 per cent. of all deaths from typhoid fever may be reckoned as due to perforation, which accordingly claims yearly about 200 victims in London and 1,500 in England and Wales. "Probably 500 lives annually might be saved if cases of perforation were recognised early and were operated on by competent surgeons of whom there is now no lack in all centres of population." Hæmorrhage is often a forerunner of perforation, but the latter accident is as liable to occur in mild and moderate as in severe cases. Males are more liable than females in the proportion of 3 to 1. Perforation is rare before the ninth or tenth day of illness. It occurs most frequently towards the end of the third and also at the beginning of the fourth week; in fact 70 per cent. of all cases occur during the second, third, and fourth weeks. During relapse the end of the second or beginning of the third week is the period of greatest liability. With regard to the site of perforation it is most common in the last 12 inches of the ileum, where ulceration is usually most severe. The small intestine may however give way higher up, but the farther from the ileo-cæcal valve the less likely is this to happen. If the large bowel gives way, a somewhat rare event, it is usually in the sigmoid flexure. In 15 per cent. there is more than one perforation. In half the cases the hole is very minute in size. Occasionally the vermiform appendix perforates. The symptoms of perforation, before peritonitis appears, demand careful study, as successful surgical treatment depends on early recognition and consequent prompt operation. Of symptoms the earliest, most important, and most constant is abdominal pain, generally more or less sudden in onset, acute, felt over the lower abdomen or referred to the right iliac region. Sometimes the pain is diffused, or it may be referred, *e.g.*, to the testicle or the end of the penis. Abdominal tenderness comes next in frequency and importance, often most marked over the lower half, especially on the right side. Vomiting, looseness of the bowels, a rigor, shock, or mental excitement also sometimes occur. The effect on temperature varies. It may cause a marked fall in some cases of pyrexia. In other cases, as when it occurs during the apyrexial period of convalescence, it may cause a rise. Change of temperature, either a rise or fall, is of some significance. The pulse usually becomes more frequent and weaker but the change may not be immediate. Where perforation is suspected the pulse should be observed every half hour. A running or thready pulse associated with other symptoms is very significant. A pinched, anxious face and a dorsal decubitus with the legs drawn up, are useful additional signs. Physical examination is very important. The abdomen should be watched daily throughout the course of typhoid fever. Softness and free respiratory mobility are favourable, distension, hardness, and immobility are of bad omen. A rigid retracted abdomen is as significant as one which is tense and distended. Liver dulness may be obliterated by the presence of free gas or by a distended colon. Leucocytosis may be some guide, but the blood examination, although useful, is not, in Mackenzie's opinion, to be too much relied upon. Leucocytosis also occurs in phlebitis, cholecystitis and other possible complications. Peritonitis without perforation, hæmorrhage, cholecystitis, perforation of the gall-bladder, and thrombosis of the iliac veins are all conditions liable to arise during typhoid fever and simulate perforation. Very careful attention must be paid to the onset and site of the pain, the condition of the patient, and the character of the physical signs. In the beginning of his lecture Mackenzie points out that the only successful treatment of perforation is prompt surgical interference. A considerable number of lives have already been saved by this means. Two cases under his own care, recently operated on by his colleague, Mr. W. H. Battle, have recovered. It is strongly advised that in all cases of typhoid fever the occurrence of perforation should be always regarded as a possible accident, that the nurse should be trained to watch for possible symptoms, and that the physician should make such arrangements as would ensure prompt surgical intervention in case of necessity. H. A. Higley [9] has made a study of the differential leucocyte count in the early days of typhoid fever. The studies of Thayer and other observers have shown that the large mono-nuclear cells are relatively, and usually absolutely, increased in numbers, while the polymorpho nuclear neutrophilic leucocytes and the eosinophiles are relatively and absolutely decreased. Thayer thought these changes were not observable before the second week, Higley, however, has observed the characteristic differential leucocyte count during the first week, but he is not yet sure of its value in aiding the early diagnosis of typhoid fever. Brown and Crompton [10] have examined 68 cases who had suffered from typhoid fever with a view to determining how long the Grüber-Widal reaction persists in the blood after recovery from this disease. The time which had elapsed since the disease occurred varied from one to 48 months in the 68 cases traced. In only three cases was a decided positive reaction obtained and in these the examination was made 2 months, 12 months, and 38 months respectively after the attack. Doubtful reactions were observed in two other cases. The test employed was absolute clumping and loss of motility in 30 minutes with a dilution of 1 in 50. All the cases tested had given this standard reaction at the time of the illness. If a dilution of 1 in 10 had been reckoned sufficient eight more cases would have given a positive reaction. J. Evans and J. Sailer [11] report a case of pure colon infection simulating typhoid fever. The blood of the patient did not give any reaction with proved cultures of the typhoid bacillus nor with four varieties of the para colon bacillus. F. G. Harris [12] strongly advocates acetozone as a remedy in typhoid fever. The preparation is similar in action to hydrogen peroxide, but the oxygen given off is in a more active condition. A solution of 12 or 15 grains to the quart is vigorously shaken and then kept in a refrigerator. It is given *ad libitum* to replace the water ordinarily given as well as in 6 ounce doses every four or six hours. The official report by Dr. Bulstrode [13] on the alleged oyster-borne epidemics of typhoid fever has been published. It deals particularly with the outbreaks following the Mayoral banquets at Winchester and Southampton. The author's conclusions are expressed with caution, but his careful investigations seem pretty clearly to implicate the oysters supplied for both those banquets from the Emsworth beds. The *Times* recently has advocated the wholesale adoption of Dr. Leigh Canney's scheme [14] for the prevention of the spread of typhoid fever among armies in the field. This consists in providing a portable system for supplying the troops with pure water. It has been pointed out, however that the discussion of such a subject by the lay public is not without its dangers The lay mind regards the dissemination of typhoid fever as a very simple matter due solely to the drinking of impure water ; to the medical mind the presence of many other contributing factors complicates the question. Pollution of milk and shellfish and especially the spread of infection by means of direct contact with the sick by means of the excreta must be carefully considered also. Hence the problem of prevention differs in rural districts from the problem as encountered in well-drained cities. The fact of infection by means of fæcal contamination, a very real source of infection, as has been shown by Koch in certain German villages, must be considered in the complicated question of preserving an army in the field from the ravages of this disease. Also the question of antityphoid inoculation in the army is not one to be lightly passed over.[15] The whole question of prevention demands the creation of a committee of sanitary experts for the purpose of working out some well-considered plan.

8 Lancet, Sept. 26. 9 Med. Rec., Sept. 19. 10 Lancet, June 27. 11 Med. Rec., July 4, p. 16. 12 Amer. Jour. Med. Sci., June, p. 1106. 13 Brit. Med. Jour., Aug. 8, p. 325. 14 Lancet, Sept. 19, p. 831. 15 Ibid, Sept. 26, p. 893.

(*To be continued.*)

